# A Study on the Impact of Ideological and Political Education of Ecological Civilization on College Students’ Willingness to Act Pro-Environment: Evidence from China

**DOI:** 10.3390/ijerph20032608

**Published:** 2023-01-31

**Authors:** Silin Liu, Lei Luo

**Affiliations:** 1 Committee of the Communist Youth League, Sichuan Agricultural University, Chengdu 611130, China; 2 College of Management, Sichuan Agricultural University, Chengdu 611130, China

**Keywords:** ecological civilization, ideological and political education, college students’ willingness to act pro-environment, econometrics model

## Abstract

The purpose of this study is to understand the impact of ideological and political education with regards to ecological civilization on college students’ pro-environment attitudes. Based on the survey data of 1622 college students, a series of econometric models are built to understand and test the impact of ideological and political education on students’ pro-environment willingness. The results show that the ideological and political education of ecological civilization can effectively promote the environmental will of college students; the accumulation of environmental knowledge, ecological concern, and ecological reflection are the important mediums for the ideological and political education of ecological civilization; and the medium effect of ecological reflection is the strongest. In addition, this study also indicates that the pro-environment willingness of college students and their professional categories, whether to join the eco-environmental association, and other factors have a significant correlation. Therefore, this study reveals the current impact of ecological civilization education and the role of the mechanism for college educators and policymakers to promote the protection of the ecological environment, which provides an important reference.

## 1. Introduction

In order to deal with the crisis of sustainable development, a growing number of policies in many countries are inclined toward the protection of ecological environments, and a large number of environmental education projects have been designed to encourage the public to engage in pro-environmental behaviour [1]. Political parties in all countries are motivated by scientific and political consideration [2], and many party leaders have included green and environmental education policies in their governing platforms. Due to environmental degradation [3], they attach great importance to university students as an important group of young people; to guide the Green Development of society through political and ideological university education, many higher education institutions are promoting the pro-environment behavior of college students through ecological education [4,5].

Non-environmental behaviors, such as excessive use of chemical substances, emission of waste gas, random disposal of waste, deforestation, and so on, aggravate global environmental pollution and ecosystem degradation [6,7,8]. In China, the problem of environmental pollution is serious; most intuitively, the amount of chemical pesticides applied per unit area of land in China is 2.5–5 times that of other developed countries [9]. However, the adoption level of green and sustainable technologies is generally low [10]. Therefore, pro-environment behavior is at a relatively low level in China, which has become one of the “bottleneck problems” restricting the sustainable development of economics and society [11]. Today, the problem of insufficient pro-environment behavior has caught the attention of the Communist Party of China, which has just concluded its twenty national congresses. The idea that “green waters and green mountains are mountains of gold and silver” must be firmly established and put into practice. Chinese leaders have also put forward the idea of an ecological civilization, believing that the construction of an ecological civilization is fundamental to the development of mankind. Consequently, the level of ecological and environmental protection has risen to an unprecedented level. We must deeply grasp the law of development between man and nature. Through political guidance, China has widely carried out ideological and political education for college students around ecological civilization [2]. This kind of education includes many kinds of forms, such as ecological knowledge education for college students through political courses, general education through campus activities, and human emotional touching and resonance. It has been found that ideological and political education about ecological civilization can effectively promote college students’ willingness to act pro-environment [12], and environmental education brings basic knowledge of environmental protection to college students [13], changing public attitudes and behaviour towards environmental protection [14]. However, what is the transmission mechanism of this effect? Is it by promoting the accumulation of knowledge by college students? Does it trigger the reflection of college students on topics of ecological concern and environmental damage? The existing research has not reached a clear conclusion; therefore, this research takes Chinese college students as an example and, through thousands of questionnaires, studies the impact of ideological and political education on ecological civilization on college students’ ecological knowledge, ecological concern, ecological reflection, and environmental behavior.

## 2. Literature Review

There is a lot of research on human’s willingness to act pro-environment, and it is generally believed that individual characteristics such as age [15], educational level [16], geographical environment [17], income [18], face consciousness [19]; and resource endowments such as family labor force [20], labor mobility [21], social learning [22], prosocial conditions [23], cultural environment [24], government policy [25,26], technology integration and application [27], and market regulation and docking [28] are related to the willingness. However, it cannot be ignored that environmental pollution is usually not easily detected, as it has hidden and scattered characteristics [29], and the impact of rigid systems of requirements and constraints on individual pro-environmental behavior is often weak [30]. In fact, it is only through education that we can generate our own green ecological consciousness and concept and strengthen the subjective initiative of individual ecological environmental protection [31]. Furthermore, this can also help us make clear the advantages and disadvantages of ecological protection and environmental destruction. Moreover, by establishing the concept of green environmental protection at the subjective level, one can increase the understanding and cognition of the ecological environment [32], which encourages people willing to carry out pro-environmental behaviors [33,34].

Studies on environmental protection education have indicated that environmental sustainability education for college students can promote the implementation of pro-environmental behavior [35,36,37], and joining campus environmental organizations can also play a promoting role [5]; formal environmental education, such as eco-environmental curriculum, has been used as one of the important channels to educate students to germinate the will of environmental protection [14]. Jurdi-hage et al. (2019) found that students’ learning of environmental knowledge, awareness, and critical thinking skills can promote their environmental literacy and sustainable lifestyles [38]. Similarly, Luo et al. (2022) used human capital theory to argue that environmental education can deepen people’s understanding of the natural environment [39]. Bradley et al. (1999) found a significant positive correlation between environmental science education and students’ pro-environment attitudes [40]. As to why ideological education can promote the use of pro-environmental behaviour by individuals, studies have suggested that education encourages students to acquire more environmental knowledge [41], which in turn promotes positive attitudes towards environmental participation [42,43]. But there are different voices: Frick et al. (2004) found that many universities, although already providing environmental knowledge, still have students who are reluctant to engage in pro-environmental behavior [44]. Kaiser and Fuhrer (2003) argue that education in ecological civilization can enhance the individual’s attention to the balance of nature and destruction [45]. Dietz et al. (2003) further suggested that responsible societies and organizations should educate individuals to increase their attention to ecological conservation, including resource systems and human-environment interactions, to understand natural variability, uncertainty, and the relative causes of environmental change and effective solutions [46]. Diaz-siefer et al. (2015) added that, at the individual level, the degree to which students pay attention to global environmental issues is strongly correlated with their pro-environmental behaviour [47]. In addition, some scholars have suggested that ecological reflection may be an important influencing factor in humans’ willingness to act pro-environment [48], but there is still a lack of empirical verification on this point.

To sum up, previous studies have focused on the impact of human pro-environmental behavior factors on....; some of the research believed that there is a certain relationship between ideological and political education of ecological civilization and college students pro-environmental behavior. However, few scholars have considered the conduction mechanism of the above-mentioned effects. Some researchers have proved that students’ knowledge accumulation and ecological concern can promote their willingness to act pro-environment, but they have not found what the intermediary role is in the influence mechanism. The main contribution of this paper is to investigate the relationship between the ideological and political education of ecological civilization and college students’ ecological knowledge, ecological concern, ecological reflection, and willingness to engage in environmentally-friendly behavior. The mediating conduction in the influence mechanism was analyzed. These results will provide a reference for the political education of eco-environmental protection in various countries and political parties.

## 3. Theoretical and Hypothesis

Human decision-making is based on the acquisition of information [49]. The theory of persuasion holds that the transmission and education of information in a particular area will promote an increase in the knowledge reserves of individuals in that area [50]. When the amount of knowledge reaches a certain level, it will change the individual’s cognitive structure and attitude to things to some extent and then affect human decision-making. For college students, the ideological and political education of ecological civilization on campus is an important way of spreading ecological and environmental knowledge, which can promote their understanding and cognition of ecological and environmental protection and generate their pro-environment will. In China, university educators can effectively carry out targeted and effective education on ecological and environmental protection knowledge, providing students with relatively new green ecological information and promoting students to construct ecological and environmental protection concepts, reducing the knowledge barrier for pro-environment behavior. In theory, the more abundant the ideological and political education in ecological civilization, the more likely the college students are to develop the concept of ecological protection, improve their understanding of the environment, and be willing to carry out pro-environmental behavior. According to this, this paper puts forward:

**Hypothesis** **1:** *The ideological and political education of ecological civilization positively affects college students’ willingness to act pro-environment.*

**Hypothesis** **2:** *The accumulation of eco-environmental knowledge plays an intermediary role in the influence of the ideological and political education of eco-civilization on college students’ willingness to act pro-environment.*

According to the theory of “Ecological Economic Man”, people in the ecological economic system not only pursue the economic rationality of maximizing economic benefits, but also have the rationality of ecological concern and ecological reflection, being willing to pay attention to ecological and environmental values and reflect on environmental damage [51]. The more ideological and political education students receive about ecological civilization, the more information they receive about ecological imbalance and environmental protection benefits, and the more likely they are to pay attention to current ecological problems, the more deeply we understand the self-interest and ecology of pro-environment behavior [52], and the more likely we are to have the endogenous motivation to carry out pro-environment behavior.

**Hypothesis** **3:** *Ecological concern plays a mediating role in the influence of the ideological and political education of ecological civilization on college students’ willingness to act pro-environment.*

**Hypothesis** **4:** *Ecological reflection plays an intermediary role in the influence of the ideological and political education of ecological civilization on college students’ willingness to act pro-environment.*

**Figure 1:**
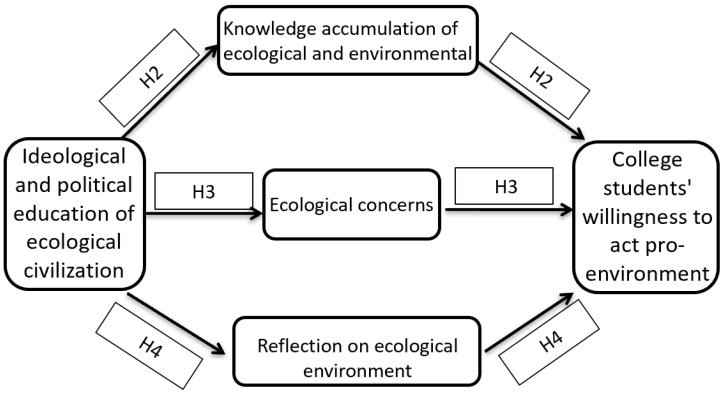
A theoretical model of college students’ willingness to act pro-environment.

According to the above theoretical analysis, the ideological and political education of ecological civilization is helpful to promote the accumulation of ecological and environmental protection knowledge, ecological concern, and ecological reflection in college students. The improvement of their knowledge, concern, and reflection can promote their willingness to engage in pro-environmental behavior. According to the logical deduction, eco-education can promote their willingness to engage in pro-environmental behavior through three mediating transmission mechanisms (Figure 1).

## 4. Data, Variables, and Descriptive Statistics

### 4.1. Data Sources

The questionnaire was released by the Communist Youth League Committee of Sichuan Agricultural University in December 2022. We adopted the method of random sampling to investigate undergraduates studying at Sichuan Agricultural University from different provinces in China and then recruited their classmates as second-level volunteers. After repeated sampling and recruitment, a total of 1785 undergraduates participated in the final questionnaire survey. Of the 1785 completed and returned questionnaires, 163 were deleted due to lack of key information and other reasons; finally, 1622 valid questionnaires were obtained, with an effective rate of 90.87%. The investigation includes the ideological and political education of ecological civilization, college students’ knowledge of ecological protection, ecological concern, ecological reflection, willingness of pro-environment behavior, individual characteristics, and family endowment, etc.

### 4.2. Variable Selection

#### Explained Variables

The questionnaire was “are you willing to perform pro-environment behavior in your life?” to indicate the willingness of the sample to perform pro-environment behavior. The results showed that the mean of the environmental behavior willingness of the sample was 4.060. This indicates that the willingness of pro-environment behavior in college students is at a high level.

### 4.3. Explanatory Variables

The questionnaire indicates the level of ideological and political education of college ecological civilization by “the number of times you receive ideological and political education of college ecological civilization in 2022”. The statistical results show that the average number of times the sample receives the ideological and political education of ecological civilization is 2.477, which indicates that the ideological and political education of ecological civilization is relatively abundant in China’s universities.

### 4.4. Mediating Variables

According to the previous theoretical analysis, this study analyzed the above-mentioned impact of ecological and environmental knowledge accumulation, ecological concern, and ecological reflection on the three transmission mechanisms, so the intermediary variables include: the accumulation of ecological and environmental protection knowledge, ecological concern, and ecological reflection. The questionnaires were presented with the following questions: “What is your knowledge of the ecological environment?” “Do you care about the problems of the ecological environment’s destruction?” “Do you reflect on the relationship between the ecological environment destruction and the human social behavior?” Assign the sample answers “Little,” “Less,” “General,” “More,” and “Many” to values 1–5, respectively. The statistical results showed that the average values of ecological knowledge accumulation, ecological concern, and ecological reflection were 2.998, 3.436, and 3.818, respectively, indicating that college students have a high level of concern and reflection on eco-environmental issues.

### 4.5. Control Variables

Referring to the existing studies [15,16,17,18,19,20,21,22,23,24], the control variables mainly include individual characteristics and family endowment. This paper controls 11 variables, such as gender, age, years of education, subject categories, etc. The exact meaning and assignment of all variables are shown in Table 1.

## 5. Method

### 5.1. Oprobit Model

For the reason that the explained variables are 1–5 ordinal variables, the Oprobit model is used to estimate the impact of ideological and political education regarding ecological civilization on college students’ willingness to act pro-environment. The empirical model is set as follows:

(1) $$willingness_{i}=\alpha_{0}+\alpha_{1}education_{i}+\alpha_{2}X_{i}+\mu_{i}$$

Among them, $willingness_{i}$ to be pro-environment behavior of the first college student, $education_{i}$ for ideological and political education of ecological civilization, $X_{i}$ for a series of control variables, including the sample of individual characteristics, family characteristics, etc. $\mu_{i}$ is a random interference term. Assuming the μ~N(0, 1) distribution, the Oprobit model can be expressed as:

$$P(willingness=1|x)=P(willingness^{*}\leq r_{0}|x)=\varphi \left(r_{0}-\alpha_{1}education_{i}-\alpha_{2}X_{i}\right)$$

$$P(willingness=2|x)=P(r_{0}<willingness^{*}\leq r_{1}|x)=\varphi \left(r_{1}-\alpha_{1}education_{i}-\alpha_{2}X_{i}\right)-\varphi \left(r_{0}-\alpha_{1}education_{i}-\alpha_{2}X_{i}\right)$$

(2) $$P(willingness=5|x)=P(r_{3}\leq willingness^{*}|x)=1-\varphi \left(r_{3}-\alpha_{1}education_{i}-\alpha_{2}X_{i}\right)$$

In (2), $r_{0}\;$<$\;r_{1}\;$<$\;r_{2}\;$<$\;r_{3}$ is the parameter to be estimated, and the value of $willingness_{i}$ is 1–5, which indicates the attitude from “unwilling” to “very willing”. By constructing the likelihood function, the parameters of the model can be estimated by maximum likelihood.

### 5.2. Instrumental Variable Method

It is difficult to avoid the conclusion that the relationship between the ideological and political education of ecological civilization and the students’ willingness to act pro-environment may lead to endogenous problems because of reverse causality and missing variables. Therefore, in order to solve the problem of estimation bias, this paper adopts the Conditional Mixed Process Method (CMP) to modify the model estimation results, thus obtaining a consistent, unbiased estimation. Based on the selection condition that the instrumental variable is highly related to the endogenous explanatory variable but not to the disturbance item, the students around the sample are selected as the instrumental variable of the model. Due to the herding effect, the individual’s level of receiving ideological education may be influenced by the education of others around him, however, there was no direct correlation between the educational status of the students around them and the sample’s willingness to act pro-environment. In theory, the instrumental variable was selected to meet the requirements of correlation and externality [53], and then two regression models were constructed to test it. It is significantly correlated with the sample of ideological education, and the test of the correlation coefficient proves that the setting of instrumental variables is reasonable.

### 5.3. The Mediation Effect Model

In order to further verify whether college students’ ecological knowledge accumulation, ecological concern, and ecological reflection play a significant intermediary role between the ideological and political education of ecological civilization and the will of pro-environment behavior. Referring to the mediation effect test method [54], the mediation effect model is set as follows:

(3) $$Y_{ki}=\alpha_{0}education_{i}+\beta_{0}X_{i}+\mu_{0}$$

(4) $$cognition_{i}=\alpha_{2}education_{i}+\beta_{2}X_{i}+\mu_{2}$$

(5) $$Y_{ki}=\alpha_{3}education_{i}+\beta_{3}cognition_{i}+X_{0}X_{i}+\mu_{3}$$

Among them, $\alpha_{0}$ in (3) reflects the total effect of ideological and political education on ecological civilization on college students’ willingness to act pro-environment, and $\alpha_{2}$ in (4) indicates the effect of ideological and political education of ecological civilization on intermediary variables, $cognition_{i}$, for the accumulation of ecological and environmental protection knowledge, ecological concern, and ecological reflection. In Formula (5), $\alpha_{3}$, $\beta_{3}$ indicate the direct effects of ideological and political education of ecological civilization and intermediary variables on the first college students’ willingness to act pro-environment. The mediating effect $\alpha_{2}\beta_{3}$ can be obtained from the Formula (4) and the substituting Formula (5), which is the indirect effect of ideological education on college students’ pro-environment behavior intention through three mediating variables. At the same time, the ratio of the mediating effect to total effect is used to reflect the relative size of the mediating effect, that is $\;\alpha_{2}\beta_{3}$/$\alpha_{0}$.

## 6. Results

### 6.1. External Driving Role of Publicity and Education

A collinearity diagnosis was performed before regression. The results showed that the variance expansion factor (VIF) of each variable was 1.02–1.61, and the mean VIF was not 1.23, which was far less than 10, indicating that there was no obvious multicollinearity problem among the variables. In Table 2, the model (1) examines the direct effect of the ideological and political education of ecological civilization on college students’ willingness to act pro-environment. The results show that ideological education has a significant positive effect on college students’ willingness to act pro-environment, and it is significant at the 1% level. The results of Model (2) further show that, on the basis of controlling the characteristics of individual sex, age, political identity, and participation in the Environmental Protection Society and Family Resource Endowment, the significant positive effect of ideological and political education of ecological civilization on college students’ willingness to act pro-environment still holds, which indicates that the higher the frequency of ideological and political education of ecological civilization, the more likely college students are to have the willingness to act pro-environment. This result is consistent with the previous theoretical conclusions.

In order to control the relationship between the ideological and political education of ecological civilization in university institutions and the students’ willingness to act pro-environment, it may lead to endogenous problems because of the reverse causality and the omission of variables; in model (3) the CMP method was used to modify the estimated results of the model. After the IV-Oprobit model was used to estimate, the coefficient of ideological and political education of ecological civilization was significantly positive, the positive effect of ideological and political education of ecological civilization on college students’ willingness to act pro-environment was tested again. The lnsig_2 value of the model was 1.256, which was significant at the level of 1%, indicating that the two-stage estimation of the model was significant, the model atanhrho_12 value was −0.552, which was significant at the 1% level. The likelihood ratio test and Atanhrho test were used to prove that the CMP method was superior to OPROBIT estimation in this model and that the use of instrumental variables was effective. Model (4) is the result of marginal effect estimation. From the result, after controlling the endogeneity problem, the effect of ideological and political education on ecological civilization on college students’ willingness to act pro-environment is similar in direction and significance to the baseline regression results reported by Model (1)(2), which verifies the positive effect. The results showed that for every unit increase in ecological civilization ideological and political education, the probability of college students’ “very willing” to participate in agricultural pro-environment behavior increased by 4.8%. Hypothesis 1 was proved.

For other control variables, gender, political status, physical health status, participation in environmental protection communities, subject category, geographical location, and family members’ work all had significant effects on pro-environment behavior willingness in different degrees, and the effects were significant at a 1–10% level. On the individual characteristics, the influence of gender on college students’ willingness to engage in pro-environment behavior is positive at the level of 1%, and the probability that women are very willing to engage in pro-environment behavior is 7.1% higher than that of men; the results show that the female youth group is more eco-rational and easier to carry out pro-environment behaviors, and the political status of party members has a positive effect on the willingness of college students to engage in pro-environment behaviors at the level of 1%. Members of the Communist Party of China are the representatives of universities who have a sense of the overall situation and advanced thinking. They attach more importance to ecological and environmental protection and usually play a leading role in demonstration on campus; the better an individual’s health condition, the more energy he or she can provide to carry out environmental protection. The 5% level of participation in environmental groups has a positive impact on college students’ behavior willingness. The possible reasons are: students learn more ecological knowledge by participating in the activities of environmental groups, which is more likely to lead to reflection on environmental protection behavior; Interestingly, subject categories also affected students’ willingness to engage in pro-environmental behaviors. Students in the humanities and social sciences were 9.3% more likely to engage in pro-environmental behaviors than students in the natural sciences; this may be because students in the humanities and social sciences receive more information about human society, think more actively, and look at problems in a more critical and reflective way. In terms of family endowment, the influence of geographical location on college students’ willingness to engage in pro-environment behaviors is significant at the 10% level. Students who live in rural areas are more likely to engage in pro-environment behaviors because of the direct contact between rural areas and the natural environment; whether or not family members engaged in environmental work contributed to the nature balance of college students at the 5% level, the more family members that individuals had directly engaged in environmental protection, the more likely they were to engage in environmental work and the more likely they were to be affected by social networks, the more likely they were to engage in pro-environmental behaviour.

### 6.2. Test of Mediating Effect of Knowledge Accumulation of Ecological and Environmental Protection

In order to confirm that the accumulation of college students’ ecological and environmental protection knowledge played the role of intermediary in the above-mentioned impact, in this paper, the author analyzes the mediating mechanism of the ideological and political education of ecological civilization on the influence of college students’ pro-environment behavior through changing the accumulation of ecological and environmental protection knowledge, tests hypothesis 2, and analyzes it with the stepwise regression method; the results are shown in Table 3. By using model (5) to analyze the relationship between ideological and political education of ecological civilization and the accumulation of ecological and environmental protection knowledge of college students, it is found that ideological and political education of ecological civilization can significantly promote the accumulation of knowledge, with a coefficient of 0.046, and pass the significance test at the level of 1%. Using the model (6) to examine the influence of college students’ environmental knowledge accumulation on their willingness to act pro-environment, the results show that, removing the variables of ideological and political education of ecological civilization, college students’ environmental knowledge accumulation also has a significant positive effect on their willingness to act pro-environment at the 1% level, indicating that the more eco-environmental knowledge students accumulate, the more likely they are to have the willingness to act pro-environment. In Model (7), the self-variable and intermediary variable are introduced, and the estimated coefficients of ideological and political education of ecological civilization and the accumulation of ecological and environmental protection knowledge of college students are both positive. Further analysis shows that the marginal effect of ideological and political education of ecological civilization on college students’ will is 0.017, and the marginal effect of eco-civilization ideological and political education is 0.026 less than that without the knowledge of eco-environmental protection, which indicates that the knowledge of eco-environmental protection plays a part of the intermediary effect. This shows that in the process of the influence of ideological and political education of ecological civilization on college students’ willingness to act pro-environment, the accumulation of ecological and environmental protection knowledge plays a part of intermediary role. Specifically, with the deepening frequency of ideological and political education in ecological civilization, the level of college students’ ecological environmental protection knowledge accumulation is also increasing, and the probability of their willingness to participate in pro-environment behavior is also increasing accordingly. The results of the mediation effect test prove that hypothesis 2 is valid.

### 6.3. Test for Mediating Effects of Ecological Concerns

In order to confirm that the ecological concern of college students in the theoretical analysis plays the role of intermediary in the above-mentioned impact, this paper deeply analyzes the intermediary transmission mechanism of the ideological and political education of ecological civilization on the influence of college students’ environmental-friendly behavior by raising their ecological concern and tests hypothesis 3 by using the stepwise regression method; the results are shown in Table 4. Using Model (8) to analyze the relationship between ideological and political education of ecological civilization and college students’ ecological concern, it is found that ideological and political education of ecological civilization has a significant promoting effect on ecological concern, with a coefficient of 0.037, and passes the significance test at the level of 1%. Using the model (9) to examine the influence of college students’ ecological concern on their willingness to act pro-environment, the results show that if the variables of ideological and political education of ecological civilization are removed, the more ecological concerns, the more likely college students are to have the intention of pro-environment behavior. In the model (10), the self-variable and the intermediary variable were introduced, and the estimated coefficients of both the ideological and political education of ecological civilization and the ecological concern of college students were positive, and the effects on the willingness of pro-environment behavior were significant at the levels of 10 and 1%, respectively; further analysis shows that the marginal effect of ideological and political education of ecological civilization on college students’ will is 0.015, which is lower than the corresponding marginal effect of ideological and political education of ecological civilization 0.026 when ecological concern is not introduced, which shows that ecological concern plays a part in the intermediary effect. This shows that ecological concern plays a part of intermediary role in the process of ecological civilization education affecting college students’ willingness to act pro-environment. Specifically, with the deepening of the frequency of ideological and political education of ecological civilization, the level of ecological concern of college students is also increasing, and they are more willing to participate in pro-environment behavior. The above-mentioned test results prove that hypothesis 3 is valid.

### 6.4. Test of Intermediary Effect of Ecological Reflection

To confirm that the ecological reflection of college students proposed in the theoretical analysis plays the role of intermediary in the above-mentioned impact, this paper analyzes the mediating mechanism of the ideological and political education of ecological civilization on the influence of college students’ pro-environment behavior by changing their ecological reflection, and it also tests hypothesis 4 by using a stepwise regression method; the results are shown in Table 5. By using model (11), this paper makes a regression analysis on the ecological civilization’s ideological and political education and the ecological reflection of college students and finds that the ecological civilization education has a significant promoting effect on ecological reflection, with a coefficient of 0.024, and passes the significance test at the level of 1%. Using Model (12) to examine the impact of college students’ ecological reflection on their willingness to act pro-environment, the results show that, after removing the variables of ideological and political education of ecological civilization, the more ecological reflection, the more likely college students are to have the intention of pro-environment behavior. The results show that the more ecological reflection, the more likely college students are to have the intention of pro-environment behavior. In Model (13), the self-variable and intermediary variable were introduced, and the estimated coefficients of ideological and political education of ecological civilization and ecological reflection of college students were both positive, and the effect on the willingness of pro-environment behavior was significant at the level of 1%; further analysis shows that the marginal effect of education about ecological civilization on college students’ will is 0.013, which is lower than the corresponding marginal effect of ideological and political education of ecological civilization 0.026 without ecological reflection, it shows that ecological reflection plays a part of intermediary effect. This shows that ecological reflection plays a part of intermediary role in the process of the influence of ecological civilization ideological and political education on college students’ willingness to act in accordance with pro-environment behavior. Specifically, with the deepening of the frequency of education on ecological civilization, the level of ecological reflection of college students is also improving, and the probability of participating in pro-environment behavior is also increasing. The results of the mediation effect test prove that hypothesis 4 is valid.

### 6.5. Robustness Check

In order to further ensure the reliability of the research conclusions, this paper tests the sample robustness of the main effect model from the aspects of samples and methods.

On the one hand, the sample robustness test is carried out. For the reason that the discussion is about the influence of the ideological and political education of ecological civilization on college students’ willingness to act pro-environment, for some college students who practice outside school, their willingness to act pro-environment has a weak correlation with the education of the university. Therefore, let’s get rid of this sample and recalculate; the results are still significant at the 1% significance level (see Table 6 for details), indicating that the robustness of the sample is good.

On the other hand, we test the robustness of the mediation test. The substitution mediation effect test was performed using the Sobel method and the Bootstrap method [55]. Different from the causal stepwise regression test, the academic circle has proposed that the product coefficient method has a better statistical effect than the causal stepwise regression method, which is gradually favored by researchers. The principle is to test whether a*b is significant. The product of the coefficients method can be divided into two categories: one is the Sobel test method, whose sampling distribution is normal based on the mediation effect; the other is Bootstrap sampling method, whose sampling distribution is non-normal based on the mediation effect. The results showed that the mediating effects of ecological knowledge accumulation, ecological concern, and ecological reflection were significant at the level of 1%. The results showed that the accumulation of ecological knowledge, ecological concern and ecological reflection played a part in mediating the above-mentioned effects, and the mediating effects accounted for 12.43, 18.17, and 35.29%, respectively. The robustness of the three mediating transmission mechanisms is verified, and the mediating effect of ecological reflection is found to be the greatest.

## 7. Discussion

### 7.1. Comparison with Existing Studies

It is of great significance to study the positive effect of ideological and political education on ecological civilization against the background of global environmental pollution. The empirical results of this paper confirm that ideological and political education about ecological civilization can effectively promote the pro-environment will of college students [56], and the deeper people’s learning and understanding of ecological civilization, the more they will be motivated to act on environmental protection [57], and ecological education in educational institutions can promote human ecological environmental behavior [58]. This study extends and expands on the preceding literature. Different from previous studies on the intention of human pro-environment behavior, this study constructs a theoretical framework based on knowledge, concern and reflection to analyze the driving and influencing mechanism of ideological and political education of ecological civilization on the environmental willingness of college students. The study finds that the accumulation of ecological and environmental protection knowledge of college students will promote the production of pro-environment behavior. A deeper understanding of environmental issues can increase the likelihood of individuals participating in environmentally friendly actions [45,59]. Oguz et al. ‘s study also supports this finding [60], but it is inconsistent with Diamantopoulos’ (2003) conclusion that a high level of environmental knowledge does not necessarily lead to a positive environmental attitude [61], which may be because with the gradual improvement and enrichment of university education, the education of ecological knowledge is more effective. This paper also found that the higher the level of ecological concern and ecological reflection, the more motivated individuals will be to act on the environment in a more responsible way, which is consistent with the research conclusion of Dietz et al. (2003) [46]. In other words, those who pay proper attention to, understand and reflect on environmental problems, damage causes and potential impacts are more willing to be responsible for the environment, which again verifies Diaz-Siefer et al. (2015) that students’ attention to global environmental issues is closely related to their pro-environmental behaviors [47].

### 7.2. Innovative Findings of This Study

Different from the above literature, the marginal contribution of this paper lies in finding instrumental variables, using the CMP method to control the bidirectional causality and the endogeneity of missing variables between ecological education and the intention of pro-environment behavior, and bringing the three transmission pathways of knowledge accumulation, ecological concern, and ecological reflection into the analysis framework. It is concluded that knowledge accumulation, ecological concern, and ecological reflection are the important mediators of the above influences. It is proposed that university education can promote the formation of pro-environment behavior by improving the knowledge accumulation, ecological concern, and ecological reflection of college students. This paper also finds that participation in environmental protection associations positively affects college students’ behavioral intentions. The possible reason is that students gain more ecological knowledge when participating in environmental protection associations, which is more likely to trigger reflection on environmental protection behaviors. What is more interesting is that the subject category also affects students’ willingness to conduct pro-environmental behaviors. Students in the humanities and social sciences have a higher probability of carrying out pro-environmental behaviors than those in the natural sciences. This may be because students in the humanities and social sciences receive more information about human society, have more active thinking, and view problems more critically and reflectively.

### 7.3. Countermeasures and Suggestions

Therefore, university educational institutions should strengthen ideological and political education on ecological civilization, strengthen education on natural ecological environment protection for college students, promote young people to understand “human and nature community”, explain the evolution relationship between human society and ecological environment from dialectical thinking, help college students to strengthen the accumulation of ecological and environmental protection knowledge, and guide them to implement pro-environment behavior.

### 7.4. Research Deficiencies and Future Directions

In addition, the deficiency of this study is that it does not examine the intrinsic motivation of students to protect the environment at a psychological level. How to mobilize and stimulate the inner thoughts of students, develop differentiated education programs according to the categories of students, encourage ecological education for college students majoring in natural sciences, and encourage college educators and decision makers to promote ecological civilization education may be hot research directions in the future.

## 8. Conclusions

This paper systematically explains the correlation mechanism between ideological and political education of ecological civilization and college students’ pro-environment behavior intention, measures the effect of ideological and political education of state civilization on college students’ pro-environment behavior intention, and demonstrates the mediating role of environmental knowledge accumulation, ecological concern, and ecological reflection. Using an econometric model and based on the survey data of 1622 college students, the empirical study found that ideological and political education of ecological civilization can effectively promote the environmental intention of college students, and the accumulation of environmental knowledge, ecological concern, and ecological reflection are important mediating channels of ideological and political education of ecological civilization, among which ecological reflection has the strongest mediating effect. In addition, this study also innovatively found that there was a significant correlation between the pro-environment intentions of college students and their major category and whether they joined the ecological environment association. Therefore, this study reveals the impact and mechanism of current ecological civilization education and provides an important reference for university educators and decision makers to promote ecological environmental protection.

## Figures and Tables

**Table 1:** Meaning and assignment of variables.

Variable Type	Variable		Assignment	Mean Value	Standard Deviation	Minimum Value	Maximum Value
Dependent variable	College students’ willingness to act pro-environment		Reluctant = 1; less willing = 2; generally = 3; more willing = 4; more willing = 5	4.060	1.017	1	5
Independent variable	Ideological and political education of ecological civilization		In 2022, the sample received the number of times of ideological and political education of ecological civilization	2.477	4.123	0	58
Intermediate variable	Knowledge accumulation of ecological and environmental protection		Very few = 1; Less = 2; generally = 3; more = 4; many = 5	2.998	0.813	1	5
	Ecological concerns		Very few = 1; Less = 2; generally = 3; more = 4; many = 5	3.436	0.908	1	5
	Ecological reflection		Very few = 1; Less = 2; generally = 3; more = 4; many = 5	3.818	0.981	1	5
Control variable	Personal characteristics of interviewees	Sex	Female = 1; Male = 0	0.313	0.464	0	1
		Age	Actual age/year	18.848	1.038	17	26
		Level of education	Actual years of education	13.039	1.078	12	19
		Political identity	Member of the Communist Party of China: Yes = 1; No = 0	0.092	0.289	0	1
		Health condition	Bad = 1; worse = 2; General = 3; Better = 4; good = 5	3.806	0.817	1	5
		Participation of environmental groups	Whether to join the eco-environmental Student Association: YES = 1; No = 0	0.113	0.317	0	1
		Subject categories	The major belongs to which classification: Humanities and Social Sciences = 1; natural sciences = 0	0.436	0.496	0	1
	Household Resource Endowment	Social Networking	Whether there are civil servants at home: Yes = 1; No = 0	0.208	0.406	0	1
		Geographical location	Whether the family is in town or rural: rural = 1; urban = 0	0.386	0.487	0	1
		Total household income	Total household income/yuan	110,390.600	151,214.300	0	2,000,000
		Family members work	Whether there is anyone in the family engaged in environmental protection work: YES = 1; No = 0	0.098	0.297	0	1

**Table 2:** The impact of ideological and political education of ecological civilization on college students’ willingness to act pro-environment.

Variable	Model (1) Oprobit	Model (2) Oprobit	Model (3) IV-Oprobit	Model (4) Marginal Utility
Ideological and political education of ecological civilization	0.052 *** (0.007)	0.026 *** (0.008)	0.148 *** (0.016)	0.048 *** (0.005)
Sex	—	−0.184 *** (0.060)	−0.217 *** (0.059)	−0.071 *** (0.019)
Age	—	−0.078 (0.034)	−0.046 (0.034)	−0.015 (0.011)
Level of education	—	−0.044 (0.032)	−0.031 (0.032)	−0.010 (0.010)
Political identity	—	0.953 *** (0.130)	0.523 *** (0.143)	0.171 *** (0.046)
Health condition	—	0.277 *** (0.035)	0.265 *** (0.035)	0.086 *** (0.011)
Participation of environmental groups	—	0.188 ** (0.096)	0.144 ** (0.102)	0.047 ** (0.033)
Subject categories	—	0.525 *** (0.060)	0.286 *** (0.069)	0.093 *** (0.022)
Social Networking	—	0.085 (0.074)	−0.136 (0.077)	−0.044 (0.025)
Geographical location	—	0.171 *** (0.062)	0.001 * (0.065)	0.000 * (0.021)
Total household income	—	0.000 (0.000)	0.000 (0.000)	0.000 (0.000)
Family members work	—	0.350 *** (0.115)	0.238 ** (0.113)	0.077 ** (0.037)
Sample size	1622	1622	1622	1622
Pseudo R^2^/lnsig_2	0.014	0.082	1.256 *** (0.018)	—
LRchi^2^/ atanhrho_12	54.24 (0.000)	319.09 (0.000)	−0.552 *** (0.080)	—
Log likelihood	−1923.62	−1791.196	−6106.218	—

Note: ***, ** and * represent the significance level at 1, 5 and 10% respectively, respectively. Figures in parentheses are robust standard errors of the coefficients. Data in the table are rounded to the nearest one. lnsig_2 is the significance test value of the second-order estimating equation, atanhrho_12 is the correlation test of the error term of the first-order estimating equation and the second-order estimating equation.

**Table 3:** Test of mediating effect of knowledge accumulation of ecological and environmental protection.

Variable	Model (5) Knowledge Accumulation of Ecological and Environmental Protection (Oprobit)	Model (6) Willingness to Act Pro-Environment (Oprobit)	Model (7) Willingness to Act Pro-Environment (Oprobit)
Ideological and political education of ecological civilization	0.046 *** (0.008)	—	0.017 * (0.008)
Knowledge accumulation of ecological and environmental protection	—	0.626 *** (0.040)	0.621 *** (0.039)
Control variables	Under control	Under control	Under control
Number of samples	1622	1622	1622
LRchi^2^	158.93 (0.000)	572.58 (0.000)	573.40 (0.000)
Loglikelihood	−1730.597	−1664.449	−1664.040

Note: ***, ** and * represent the significance level at 1, 5 and 10% respectively.

**Table 4:** Test for mediating effects of ecological concerns.

Variable	Model (8) Ecological Concerns (Oprobit)	Model (9) Willingness to Act Pro-Environment (Oprobit)	Model (10) Willingness to Act Pro-Environment (Oprobit)
Ideological and political education of ecological civili-zation	0.037 *** (0.008)	—	0.015 * (0.008)
Ecological concerns	—	0.796 *** (0.038)	0.792 *** (0.038)
Control variables	Under control	Under control	Under control
Number of samples	1622	1622	1622
LRchi^2^	161.13 (0.000)	798.47 (0.000)	798.87 (0.000)
Loglikelihood	−1913.833	−1551.506	−1551.3047

Note: ***, ** and * represent the significance level at 1, 5 and 10% respectively.

**Table 5:** Test of intermediary effect of ecological reflection.

Variable	Model (11) Ecological Reflection (Oprobit)	Model (12) Willingness to Act Pro-Environment (Oprobit)	Model (13) Willingness to Act Pro-Environment (Oprobit)
Ideological and political education of ecological civili-zation	0.024 *** (0.008)	—	0.013 *** (0.008)
Ecological reflection	—	0.663 *** (0.033)	0.659 *** (0.033)
Control variables	Under control	Under control	Under control
Number of samples	1622	1622	1622
LRchi^2^	87.41 (0.000)	735.64 (0.000)	738.34 (0.000)
Loglikelihood	−2000.613	−1582.919	−1581.570

Note: ***, ** and * represent the significance level at 1, 5 and 10% respectively.

**Table 6:** Sample robustness test.

Variable	Model (14) Oprobit	Model (15) Oprobit
Ideological and political education of ecological civili-zation	0.049 *** (0.007)	0.024 *** (0.008)
Control variables	Uncontrolled	Under control
Sample size	1555	1555
LRchi^2^	81.23 (0.000)	299.91 (0.000)
Pseudo R^2^	0.087	0.091

Note: ***, ** and * represent the significance level at 1, 5 and 10% respectively.

## Data Availability

The data presented in this study are available within the article.
